# Occurrence of Severe Arrhythmias in Patients with Non-ST Elevation Acute Coronary Syndrome (NSTE-ACS): A Retrospective Study

**DOI:** 10.3390/jcm12103456

**Published:** 2023-05-14

**Authors:** Valérie Wilmé, Sébastien Harscoat, François Séverac, Adrien Carmona, Pierrick Le Borgne, Pascal Bilbault, Olivier Morel, Sabrina Kepka

**Affiliations:** 1 Emergency Department, Hôpitaux Universitaires de Strasbourg, 67091 Strasbourg, France; 2 Public Health Department, Hôpitaux Universitaires de Strasbourg, 67091 Strasbourg, France; 3 Interventional Cardiology Department, Hôpitaux Universitaires de Strasbourg, 67091 Strasbourg, France; 4 French National Institute of Health and Medical Research (INSERM), UMR 1260, Regenerative NanoMedicine (RNM), Fédération de Médecine Translationnelle (FMTS), University of Strasbourg, 67000 Strasbourg, France; 5 ICube, UMR 7357 CNRS, 67400 Illkirch-Graffenstaden, France

**Keywords:** acute coronary syndrome, NSTEMI, unstable angina, cardiac arrhythmia, cardiac conduction defect

## Abstract

Background: Non-ST elevation acute coronary syndrome (NSTE-ACS) is one of the most frequent manifestations of coronary artery disease. The occurrence of serious heart rhythm disorders (SHRDs) in NSTE-ACS is not well documented. However, continuous heart rhythm monitoring is recommended during the initial management of NSTE-ACS. The targeted monitoring of patients at greater risk for SHRDs could facilitate patients’ care in emergency departments (EDs) where the flow of patients is continuously increasing. Methods: This retrospective single-center study included 480 patients from emergency and cardiology departments within the Strasbourg University Hospital between 1 January 2019 and 31 December 2020. The objective was to estimate the frequency of the occurrence of SHRDs among patients with NSTE-ACS. The secondary objective was to highlight the factors associated with a higher risk of SHRDs. Results: The proportion of SHRDs during the first 48 h of hospital care was 2.3% (CI95%: 1.2–4.1%, n = 11). Two time periods were considered: before coronary angiography (1.0%), and during, or after coronary angiography (1.3%). In the first group, two patients required immediate treatment (0.4% of the patients) and no death occurred. In the univariate analysis, the variables significantly associated with SHRDs were age, anticoagulant medication, a decrease in glomerular filtration rate, plasmatic hemoglobin, and left ventricle ejection fraction (LVEF), and an increase in plasmatic troponin, BNP, and CRP levels. In the multivariable analysis, plasmatic hemoglobin > 12 g/dL seemed to be a protective factor for SHRDs. Conclusions: In this study, SHRDs were rare and, most often, spontaneously resolved. These data challenge the relevance of systematic rhythm monitoring during the initial management of patients with NSTE-ACS.

## 1. Introduction

Cardiovascular disease is the leading cause of death worldwide. According to the World Health Organization (WHO), the most frequent cause of death of a cardiovascular origin is coronary artery disease, causing more than seven million deaths per year in the world and representing more than 13% of global mortality [1]. The acute expression of coronary artery disease encountered in emergency medicine is acute coronary syndrome (ACS), of which the clinical symptom is chest pain, making this frequent symptom a major diagnostic and therapeutic issue in emergency departments [2].

After performing an electrocardiogram (ECG), two groups of patients are distinguished, and the groups undergo a different management and prognosis: patients with persistent ST-segment elevation for more than 20 min (ST-segment elevation myocardial infarction, STEMI) and patients without persistent ST-segment elevation (non-ST elevation acute coronary syndrome, NSTE-ACS), including unstable angina and non-ST segment elevation myocardial infarction (NSTEMI). In NSTE-ACS, electrocardiographic changes may include transient ST segment elevation, ST segment depression (persistent or transient), and T-wave inversion or flattening, but the result of the ECG may also be normal [3]. NSTE-ACS represents more than 60% of ACS, and observations over the past decade suggest that the incidence of STEMI has decreased, while that of NSTE-ACS has increased [4,5]. Once diagnosed based on the results of the ECG, the management of STEMI involves immediate myocardial reperfusion [6]. In contrast, the management of NSTE-ACS is based on a risk assessment using scores (e.g., TIMI, ESC, GRACE, etc.), and it also depends on the measurement of cardiac biomarkers.

Complications of myocardial infarction can occur during NSTE-ACS, but these are less common compared to those associated with STEMI, including rhythm and conduction disorders potentially associated with death. Currently, few studies have investigated the frequency of the occurrence of serious rhythm and conduction disorders in NSTE-ACS. These studies are mostly related to inter-hospital transport, suggesting that the frequency of serious events detected on a cardioscope is low [7,8]. However, it is recommended to provide continuous rhythm monitoring for NSTE-ACS patients [9], which mobilizes material and human resources, both during out-of-hospital and in-hospital patient management. In the case of NSTE-ACS, this monitoring for several hours while waiting for the first biological results may cause difficulties in managing the flow of patients within the emergency department. In the context of overcrowding in these units, the study of the predictive factors associated with the occurrence of serious rhythm disorders appears to be of paramount importance to select continuous rhythm monitoring in high-risk patients.

In the present study, the authors aimed to estimate the frequency of the occurrence of serious heart rhythm disorders (SHRDs) during the first 48 h of hospital care in a population of NSTE-ACS patients admitted to emergency and cardiology departments.

## 2. Materials and Methods

### 2.1. Study Design and Population

The authors conducted a retrospective study in the emergency and cardiology departments of a university hospital in France. Data were collected between October 2021 and March 2022. The medical records used for the data collection were those of patients admitted to the emergency and cardiology departments for NSTE-ACS between January 2019 and December 2020. This explains the retrospective nature of the study, and such a design was considered appropriate to address the research question. Since patients with NSTE-ACS were identified before the study, this allowed for the study of rare events (in this case, SHRDs due du NSTE-ACS) in a shorter period of time than a prospective cohort study. A total of 500 consecutive patients aged over 18 years were admitted for NSTE-ACS, based on the International Classification of Diseases—Tenth Revision (ICD-10), codings I21.4-(NSTEMI), and I20.0 (unstable angina) to the emergency and cardiology departments of the University Hospital of Strasbourg were included.

### 2.2. End-Point Definitions

The primary objective was to estimate the frequency of SHRDs in patients with NSTE-ACS during the first 48 h of in-hospital management. The SHRDs include potentially lethal arrhythmias warranting continuous heart rhythm monitoring. Therefore, the authors used a composite primary end point corresponding to the following events: ventricular rhythm disorders (torsade de pointes, ventricular tachycardia [VT], ventricular fibrillation [VF]), high-grade conductive disorders (second-degree atrioventricular blocks [AVB] type Mobitz 2, complete AVB, and trifascicular blocks), electromechanical dissociation (EMD), and/or asystole. The occurrence of at least one of the above events was sought for each patient during the first 48 h of their hospitalization, the period accepted as when patients are at the highest risk of suffering rhythmic complications [10,11].

The secondary objective was to study the individual factors associated with the occurrence of SHRDs.

### 2.3. Data Collection

Hospitalization and monitoring data were obtained from the patients’ electronic medical records (EMRs). Using each patient’s EMRs, the following data were collected and entered into a computerized case report form: demographic and anthropometric characteristics, cardiovascular risk factors (CVRFs), comorbidities, long-term treatment, vital signs at the time of admission, characteristics of the first ECG, laboratory tests results at admission, patient’s description of a “typical” chest pain according to the European Society of Cardiology (ESC) criteria [9], initial management including whether or not a coronary angiography was performed, the SYNTAX score (SXS), and clinical SYNTAX score (CSS) values calculated from the lesions visualized during coronary angiography. These scores are predictive of the occurrence of major cardiovascular events after myocardial revascularization [12,13].

### 2.4. Statistical Analysis

All data were analyzed using the 4.0.2 version of R statistical software. The analysis consists of a descriptive and an analytical part. The descriptive analysis is univariate and bivariate, according to the presence or absence of SHRDs. For comparative analyses, a *p* value < 0.05 is considered statistically significant. For categorical variables, the analysis summarizes the variables by the number and frequency of each modality. Comparisons were made using Fischer’s exact test. The quantitative variables were described using the median with an interquartile range. Comparisons were made using Student’s *t*-test for continuous variables, or a Kruskal–Wallis test according to the normality of the variable.

The estimation of the frequency of the occurrence of SHRDs in patients with NSTE-ACS was performed by calculating the proportion of patients who presented an SHRD and the associated 95% confidence interval. The calculation of the confidence interval was performed using the Agresti–Coull method.

The identification of the factors associated with the occurrence of an SHRD was performed using the multivariable logistic regression model. First, univariate analyses between the dependent variable (occurrence of an SHRD) and all the potential associated factors were performed. For the second step, the multivariable model was constructed by integrating the variables presenting a *p*-value lower than 0.01 in univariate analysis and/or the variables of clinical interest. The results are presented as odds ratios with their 95% confidence intervals.

The number of subjects was calculated by determining the precision (width of the 95% confidence interval) of the expected estimate on the primary end point. Data from the literature suggest that complications such as conduction disorders and asystole would be in the range of 0.9 to 2.2% [14,15,16], whereas ventricular rhythm disorders would occur in 7.6 to 12.9% of NSTE-ACS [11,17]. Thus, the frequency of SHRDs during NSTE-ACS could be between 8.5% and 15.1%. However, this frequency is probably overestimated. Indeed, none of the estimates described above consider that the same patient may present several rhythmic complications. The inclusion of 400 patients allows us to estimate this frequency with a precision of 5 to 9%, depending on the value of the point estimate. This precision was considered clinically satisfactory. In order to take into account possible missing data, the number of subjects required was set at 500.

### 2.5. Ethics

This study was conducted in accordance with the principles set forth by the Good Clinical Practice guidelines and the Declaration of Helsinki. The study was approved by the Ethics Committee (CE No.-2021-117). In accordance with French legislation, formal written informed consent was not required for this type of study because the data studied were entirely retrospectively [18]. An information and non-opposition form was sent to each patient, allowing them to express a possible refusal of inclusion in the study.

## 3. Results

**Figure 1:**
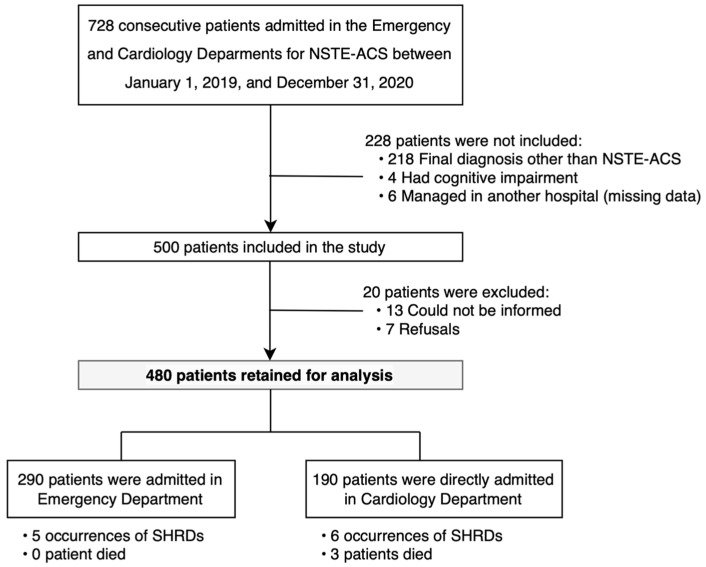
Flow chart of the study population. Abbreviations: NSTE-ACS: non-ST elevation acute coronary syndrome.

Between 1 January 2019, and 31 December 2020, 728 patients admitted to the emergency and cardiology departments were selected, and 500 met the inclusion criteria. In total, 7 patients refused the anonymized use of their medical record data, and 13 could not be informed because they did not have a valid address. The records of 480 patients were retained for the analysis (Figure 1).

### 3.1. Baseline Characteristics of the Study Population

The characteristics of the 480 patients analyzed are presented in Table 1. In this study, 330 men and 150 women were included, and the median age was 69 years (interquartile range [IQR] [59.0; 78.0]). The predominant cardiovascular risk factors (CVRFs) were hypertension (68.5%, *n* = 329) and dyslipidemia (52.1%, *n* = 250). Almost half of the patients admitted for NSTE-ACS had a history of ischemic heart disease (48.1%, *n* = 231).

Among the individual patient characteristics, only older age appeared to be associated with the occurrence of SHRDs. In contrast, the percentage of anticoagulation was statistically significantly higher in patients with an SHRD. The type of antiplatelet therapy and other cardiovascular treatments were not associated with the occurrence of SHRDs.

**Table 1:** Baseline characteristics of the study population according to occurrence or not of serious heart rhythm disorder (SHRD).

Variable		Study Population	No SHRD	SHRD	*p*-Value
		*n* = 480	*n* = 469	*n* = 11	
Individual characteristics, median [IQR]					
	Age, in years	69.0 [59.0; 78.0]	69.0 [59.0; 78.0]	85.0 [71.5; 88.5]	0.004 *
	BMI, kg/m^2^	26.46 [23.89; 30.45]	26.5 [24.1; 30.5]	23.9 [22.8; 30.2]	0.361
	Female, *n* (%)	150 (31.2)	144 (30.7)	6 (54.5)	0.175
Cardiovascular risk factors (CVRFs), *n* (%)					
	Heredity	102 (21.2)	101 (21.5)	1 (9.1)	0.532
	Smoking	125 (26.0)	124 (26.4)	1 (9.1)	0.343
	High blood pressure	329 (68.5)	321 (68.4)	8 (72.7)	1.000
	Diabetes mellitus	175 (36.5)	169 (36.0)	6 (54.5)	0.345
	Dyslipidemia	250 (52.1)	244 (52.0)	6 (54.5)	1.000
	Obesity	122 (25.4)	118 (25.2)	4 (36.4)	0.622
	Number of CVRFs, median [IQR]	2.0 [1.0; 3.0]	2.0 [1.0; 3.0]	2.0 [1.0; 3.5]	0.872
Comorbidities, *n* (%)					
	Ischemic heart disease	231 (48.1)	225 (48.0)	6 (54.5)	0.900
	Ischemic stroke	41 (8.5)	40 (8.5)	1 (9.1)	1.000
	Peripheral arterial disease	67 (14.0)	65 (13.9)	2 (18.2)	1.000
	Renal failure	81 (16.9)	77 (16.4)	4 (36.4)	0.181
	Heart failure	23 (4.8)	23 (4.9)	0 (0.0)	0.969
	Supraventricular rhythm disorder	65 (13.5)	63 (13.4)	2 (18.2)	0.993
Long-term treatment, *n* (%)					
	Anticoagulant	67 (14.0)	65 (13.9)	2 (18.2)	0.010 *
	Antiplatelet agent	243 (50.6)	237 (50.5)	6 (54.5)	1.000
	ACE inhibitor	134 (27.9)	132 (28.1)	2 (18.2)	0.698
	ARB	116 (24.2)	115 (24.5)	1 (9.1)	0.409
	Beta-blocker	237 (49.5)	233 (49.8)	4 (36.4)	0.565
	Statin	215 (44.8)	210 (44.8)	5 (45.5)	1.000

* *p* < 0.05. Abbreviations: SHRD: serious heart rhythm disorder, IQR: interquartile range, BMI: body mass index, CVRFs: cardiovascular risk factors, ACE: angiotensin-converting enzyme, ARB: angiotensin receptor blocker.

### 3.2. In-Hospital Management

The characteristics at admission and complications during initial management are summarized in Table 2. Patients were usually admitted to the emergency department (60.4%, *n* = 290), less often to the cardiology department (39.6%, *n* = 190). Being managed in the cardiology department rather than the emergency department at admission did not have a significant impact on the occurrence of SHRDs.

Typical chest pain, as described by the ESC, was reported by 55.6% (*n* = 266) of patients. The ECG at admission was abnormal in 58.1% (*n* = 279) of cases, with a majority of T-wave changes (69.5%, *n* = 194). ST segment depression was present in 25.4% (*n* = 71) of the abnormal admission ECGs, and the median sum of the depression in all leads was 5.0 mm (IQR [3.0; 6.0]). These repolarization disorders mainly involved the inferior (20.3%, *n* = 54) and extended anterior territory (15.8%, *n* = 42). Only 1.7% (*n* = 8) of patients in this study had a new onset supraventricular rhythm disorder at admission, and it was atrial fibrillation in all cases. Extrasystoles were present in 9% (*n* = 43) of patients. None of the electrocardiographic abnormalities appeared to be associated with the occurrence of SHRDs. No association was found between the coronary territory involved and the occurrence of SHRDs. As expected, the prevalence of cardiogenic shock and death was significantly higher in SHRD patients.

Regarding the initial biological characteristics, the SHRD patients’ group were more often anemic with a lower median GFR and higher plasma troponin, BNP, and CRP. LVEF was also lower in SHRD patients.

Coronary angiography was performed in 75.4% (*n* = 362) of patients within 48 h after their admission, and 79.6% (*n* = 288) of them underwent percutaneous coronary angioplasty during this procedure. Before angioplasty, an analgesic drug (excluding nitrates) was administered to 20.2% (*n* = 97) of patients. In more than two thirds of cases, the highest level of pain medication used was paracetamol (68.0%, *n* = 66) and, more rarely, a level 2 analgesic (14.4%, *n* = 14) or morphine (17.5%, *n* = 17).

### 3.3. Primary End-Point

**Figure 2:**
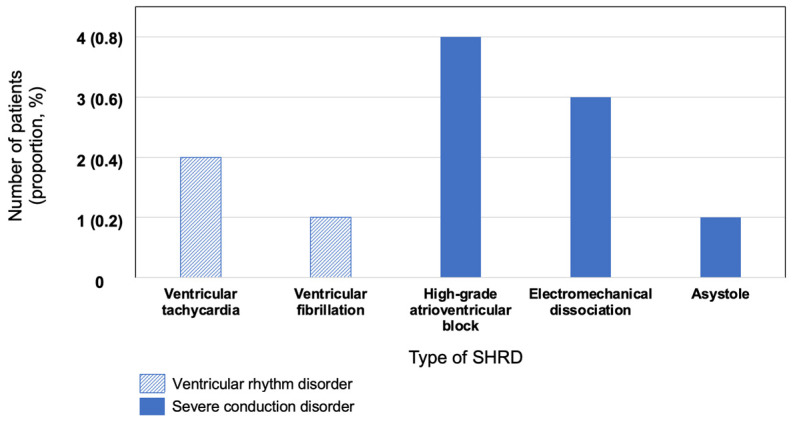
Distribution of serious heart rhythm disorders (SHRDs). Abbreviations: SHRD: serious heart rhythm disorder, CI95%: 95% confidence interval.

Among the 480 patients of the study, SHRDs occurred for 11 patients (2.3%) within the first 48 h of management, with a 95% confidence interval of [1.2%; 4.1%]. The distribution of SHRDs is presented in Figure 2, and the characteristics of these events are presented in Table 3.

Six SHRDs occurred during or after coronary angiography with angioplasty (patients N°6, 7, 8, 9, 10, and 11). This represents 1.3% of the population (95% CI [0.5%; 2.8%]) and three of these patients died after the revascularization procedure (N°6, 8, and 9). Patients N°6 and N°9 died of refractory cardiogenic shock following return of spontaneous circulation. Patient N°8 died of refractory cardiac arrest. No deaths occurred before coronary angiography. A more detailed description of the patients and events is available in Supplementary Material S1.

### 3.4. Multivariable Analysis

Variables with the lowest *p*-values (age, troponin, BNP, CRP, hemoglobin, and GFR), or those whose association with SHRDs is well documented in the literature (LVEF) were retained for the multivariable analysis (Table 4). The analysis was performed considering that the risk increases proportionally with changes in the variable of interest. The variables troponin, CRP, GFR, and hemoglobin were each analyzed using a threshold to distinguish a normal value from a pathological value. The thresholds chosen were based on the standards used in the Strasbourg University Hospital laboratories. In this study, plasmatic hemoglobin > 12 g/dL is associated with a lower risk of SHRD (OR = 0.12, 95% CI [0.02; 0.48]) and, therefore, could be considered a protective factor for SHRD in patients with NSTE-ACS.

## 4. Discussion

In this retrospective study including patients hospitalized in the emergency and cardiology departments for NSTE-ACS, 11 patients out of 480 presented an SHRD during the first 48 h of in-hospital management, which represents 2.3% of the study population (95% CI [1.2%; 4.1%]). Most of these were severe conduction disorders. The occurrence of SHRDs does not appear to differ according to the realization or not of a coronary angiography; six of these events occurred during or in the hours following coronary angiography for 1.3% of the study population (95% CI [0.5%; 2.8%]) and three of them died after inaugural electro-mechanical dissociation. An SHRD before coronary angiography concerned five patients (1.0% of the study population) (95% CI [0.4%; 2.5%]), therapeutic intervention was required for only two of them (0.4% of the study population, 95% CI [0.01%; 1.6%]), and none died during the first 48 h of in-hospital management. Among the factors studied, older age, anticoagulant medication, a decrease in LVEF, GFR, and plasma hemoglobin, and an increase in plasma troponin, BNP, and CRP were statistically associated with the occurrence of SHRD. Significantly higher troponin and BNP values are suggestive of greater myocardial involvement in the SHRD population, while the decreased LVEF observed in this population might increase the risk of myocardial infarction. After multivariable analysis, having plasmatic hemoglobin above 12 g/dL appears to be independently associated with a lower risk of SHRD occurrence.

To the best of the authors’ knowledge, this study is the first to comprehensively estimate the frequency of SHRD, including both ventricular rhythm disorders and severe conduction disorders in NSTE-ACS.

With regard to ventricular rhythm disorders, the prevalence within 48 h of inclusion was estimated at 0.6% by Piccini et al. [19] for patients presenting a high-risk NSTE-ACS. This prevalence is lower than the estimated prevalence of rhythm disorders in NSTE-ACS in other studies, which is supposed to be between 5 and 10% [19]. In the study by Gupta et al. [11], the prevalence of ventricular rhythm disorders was 7.6%, and 60% occurred in the first 48 h. For Tran et al. [17], this estimate was higher, with 11.3% of VT and 1.6% of VF, but the difference may be partly explained by the period of interest in this study, whereas it appears that a significant proportion of rhythm disorders in association with NSTE-ACS occur beyond 48 h [11,17,19]. Regarding conduction disorders and asystole, the study by Alnsasra et al. [20] included 49 patients with high-grade AVB out of 5612 with NSTE-ACS, i.e., 0.9% of the population. This estimate was 0.6% in the study by Misumida et al. [14] and 0.4% in the study by Pokorney et al. [15]. The prevalence of asystole and EMD was estimated to be 0.5% and 0.1%, respectively, in the study by Pokorney et al. [15].

The percentage of complications related to coronary angiography for all indications combined was estimated at 1.7% by Gach et al. [21], taking into account major complications of the procedure such as death, rhythm disorders, stroke, vascular complications, or hemodynamic and allergic complications.

In previous studies, advanced age, elevated plasma troponin, impaired renal function, and decreased LVEF were identified as the factors significantly associated with the occurrence of SHRDs in patients with NSTE-ACS [11,14,15,19,20]. To our knowledge, there is no data in the literature regarding the association between the occurrence of SHRDs and BNP, CRP, and hemoglobin values in patients with NSTE-ACS. The study of Sauer et al. [22] showed an increased risk of ventricular rhythm disorders with morphine administration in patients with STEMI. Furthermore, the studies of Tran et al. [23] and Liu et al. [24] suggested an increased risk of rhythm disorders with hyperglycemia in patients with NSTE-ACS.

### 4.1. Strengths and Limitations

The retrospective nature of the study and the small number of events of interest are limitations that have to be taken into account when interpreting the results. There are indeed few SHRDs, and such a small number of cases certainly led to insufficient statistical power and a biased estimate of logistic regression coefficients. The results of the univariate analysis and the multivariable model should, therefore, be cautiously interpreted.

Despite these limitations, the main strength of the study is that it was conducted with a large cohort of patients. Many potential risk factors, including clinical, biological, or electrocardiographic, were taken into account. Some of them, such as hemoglobin, have never been studied in the literature. The a priori calculation of the number of required subjects ensured a certain degree of precision in the estimate.

### 4.2. Implications

The present study provides interesting initial data for a simple estimation of the overall risk of SHRDs. These results could be confirmed and completed by a larger study to identify a population at risk. Similar work on pre-hospital and inter-hospital transport would be interesting, given their potential impact on the means to be used for transporting these patients. A precise individual assessment of the risk of SHRDs (e.g., using a prediction score) would allow for less strict but more appropriate monitoring. This could lead to new recommendations outlining the criteria for monitoring NSTE-ACS patients, instead of the current guidelines that recommend the routine monitoring of all these patients. This approach aims to make patient management more fluid, without reducing the quality and safety of care.

## 5. Conclusions

In this study, serious heart rhythm disorders (SHRDs) occurring during the first 48 h of hospital management were rare and, most often, spontaneously resolved. Currently, continuous heart rhythm monitoring for at least 24 h is recommended during the management of patients with NSTE-ACS. These recommendations are not evidence based, and, to date, no studies have addressed the prevalence of all SHRDs in NSTE-ACS. The low proportion of SHRDs challenges the value of systematic cardiac rhythm monitoring during initial management, once the diagnosis of STEMI has been eliminated.

## Figures and Tables

**Table 2:** Characteristics and complications during in-hospital management according to the occurrence of serious heart rhythm disorder (SHRD).

Variable		Study Population	No SHRD	SHRD	*p*-Value
		*n* = 480	*n* = 469	*n* = 11	
Initial admission in cardiology department, *n* (%)		190 (39.6)	184 (39.2)	6 (54.5)	0.475
Typical chest pain, *n* (%)		266 (55.6)	259 (55.5)	7 (63.6)	0.816
Vital signs at admission, median [IQR]					
	Heart rate, in beats per min (bpm)	77.0 [67.0; 89.0]	77.0 [67.0; 89.0]	80.0 [66.0; 91.5]	0.813
	Systolic BP, in mmHg	144.0 [128.0; 160.0]	144.0 [128.0; 160.0]	136.0 [125.5; 150.0]	0.295
	Diastolic BP, in mmHg	79.0 [70.0; 90.0]	79.0 [70.0; 90.0]	77.0 [68.0; 85.5]	0.396
	Pulse oximetry, in %	97.0 [96.0; 98.0]	97.0 [96.0; 98.0]	98.0 [97.5; 98.5]	0.110
	Capillary blood glucose, in mmol/L	1.19 [1.01; 1.58]	6.54 [5.55.; 8.69]	8.47 [7.09; 10.56]	0.082
	Temperature, in °C	36.6 [36.2; 37.0]	36.6 [36.2; 37.0]	36.5 [36.2; 36.8]	0.429
ECG upon admission					
	Abnormal ECG, *n* (%)	279 (58.1)	273 (58.2)	6 (54.5)	1.000
	ST segment depression, *n* (%)	71 (14.8)	69 (14.7)	2 (18.2)	1.000
	Sum of the depression, median [IQR]	5.0 [3.0; 6.0]	4.5 [3.0; 6.0]	8.5 [6.8; 10.3]	0.160
	Abnormal T waves, *n* (%)	194 (40.7)	189 (40.6)	5 (45.5)	0.987
	QTc interval, median [IQR] in ms	405.0 [380.0; 426.0]	405.0 [380.0; 426.0]	392.0 [374.5; 415.5]	0.375
	RT interval, median [IQR] in ms	260.0 [240.0; 280.0]	260.0 [240.0; 280.0]	280.0 [250.0; 320.0]	0.119
Biology upon admission, median [IQR]					
	Troponin, in ng/L	288.4 [37.1; 1562.6]	276.0 [34.9; 1525.1]	2096.1 [1180.2; 11,995.4]	0.002 *
	BNP, in ng/L	108.5 [40.0; 305.0]	107.0 [39.0; 272.0]	431.0 [270.0; 987.0]	0.002 *
	CRP, in mg/L	0.0 [0.0; 10.5]	0.0 [0.0; 9.25]	17.8 [5.4; 34.3]	0.005 *
	Hemoglobin, in g/dL	13.40 [12.0; 14.6]	13.5 [12.1; 14.7]	10.7 [10.5; 11.7]	0.001 *
	Leukocytes, in G/L	8.57 [7.03; 10.43]	8.57 [7.03; 10.44]	8.44 [7.26; 9.44]	0.848
	Platelets, in G/L	240.5 [204.0; 293.0]	240.0 [203.0; 292.0]	267.0 [224.5; 348.5]	0.078
	Mean platelet volume, in fl	10.4 [9.8; 11.0]	10.4 [9.8; 11.0]	10.9 [9.95; 11.2]	0.352
	Potassium, in mmol/L	4.0 [3.8; 4.3]	4.0 [3.8; 4.3]	4.2 [3.9; 4.3]	0.481
	Creatinine, in µmol/L	73.4 [61.8; 96.6]	75.3 [61.7; 96.0]	84.9 [72.2; 128.0]	0.102
	Urea, in mmol/L	6.4 [5.0; 8.5]	6.35 [5.0; 8.5]	7.2 [6.5; 14.9]	0.150
	GFR (CKD-EPI), in mL/min/1.73m^2^	83.5 [61.0; 98.0]	84.0 [62.0; 98.0]	63.0 [39.0; 70.5]	0.005 *
LVEF, in %, median [IQR]		57.0 [50.0; 64.0]	58.0 [50.0; 64.0]	47.0 [42.0; 56.0]	0.028 *
Initial management, *n* (%)					
	Coronary angiography	362 (75.4)	354 (75.5)	8 (72.7)	1.000
	Coronary angioplasty	288 (60.0)	280 (59.7)	8 (72.7)	0.575
	SXS, median [IQR]	7.0 [0.0; 14.0]	7.0 [0.0; 14.0]	13.0 [4.5; 19.0]	0.160
	CSS, median [IQR]	8.1 [0.0; 22.5]	8.0 [0.0; 22.2]	33.0 [6.8; 41.7]	0.066
	Oxygen therapy	74 (15.4)	71 (15.1)	3 (27.3)	0.497
	Analgesic	97 (20.2)	96 (20.5)	1 (9.1)	0.781
Early complications, *n* (%)					
	STEMI	1 (0.2)	1 (0.2)	0 (0.0)	1.000
	Cardiogenic shock	4 (0.8)	1 (0.2)	3 (27.3)	<0.001 *
	Acute pulmonary oedema	17 (3.5)	16 (3.4)	1 (9.1)	0.855
	Non-life-threatening rhythm disorder	64 (13.3)	62 (13.2)	2 (18.1)	0.229
Death		3 (0.6)	0 (0.0)	3 (27.3)	<0.001 *

* *p* < 0.05. Abbreviations: SHRD: serious heart rhythm disorder, IQR: interquartile range, bpm: beats per minute, BP: blood pressure, ECG: electrocardiogram, ms: milliseconds, BNP: B-type natriuretic peptide, CRP: C-reactive protein, GFR: glomerular filtration rate, LVEF: left ventricle ejection fraction, SXS: SYNTAX score, CSS: clinical SYNTAX score, STEMI: ST elevation myocardial infarction.

**Table 3:** Specific characteristics related to the occurrence of serious heart rhythm disorder (SHRD).

Patient	Type of SHRD	Symptom	Persistent Chest Pain	Time of Occurrence in Relation to Coronary Angiography	Therapeutic Measure	Ionic Disorders	Death
N°1	2nd-degree AVB type Mobitz II (transient, 10 s)	No	No	Before coronary angiography, nocturnal occurrence	No	Yes, hypokalemia 2.8 mmol/L	No
N°2	Ventricular tachycardia	No	No	Before coronary angiography	No	No	No
N°3	3rd-degree AVB	Faintness	No	Before coronary angiography	Non-invasive temporary cardiac pacing	No	No
N°4	Ventricular tachycardia	CRA	No	Before coronary angiography	1 EEC, amiodarone	No	No
N°5	3rd-degree AVB	No	No	Before coronary angiography, nocturnal occurrence	No	No	No
N°6	EMD	CRA	Yes	In catheterization room, after angioplasty	CPR	Yes, hypocalcemia 1.52 mmol/L (with QTc = 519 ms)	Yes
N°7	Ventricular fibrillation	CRA	No	In catheterization room, after angioplasty	1 EEC	No	No
N°8	EMD	CRA	No	In catheterization room, after angioplasty	CPR	No	Yes
N°9	EMD	CRA	No	5 h after coronary angioplasty	CPR	No	Yes
N°10	Asystole (transient, <30 s)	Syncope	No	9 h after coronary angioplasty	ECM, amiodarone	No	No
N°11	3rd-degree AVB	No	No	15 h after coronary angioplasty, nocturnal occurrence	Isoprenaline	No	No

Abbreviations: SHRD: serious heart rhythm disorder, AVB: atrioventricular block, s: seconds, CRA: cardiorespiratory arrest, EEC: external electric shock, EMD: electro-mechanical dissociation, CPR: cardiopulmonary resuscitation, h: hours, ECM: external cardiac massage.

**Table 4:** Multivariable analysis of factors associated with serious heart rhythm disorder occurrence.

Variable	OR	CI95%	*p*-Value
Age ^a^, in years	1.69	[0.96; 3.32]	0.073
LVEF ^a^, in %	0.86	[0.52; 1.43]	0.569
Troponin > 37 ng/L	4.87	[0.53; 649.67]	0.195
BNP ^b^, in ng/L	0.98	[0.88; 1.04]	0.471
CRP > 4 mg/L	2.60	[0.69; 11.73]	0.159
GFR < 60 mL/min/1.73m^2^	0.81	[0.20; 3.01]	0.749
Hemoglobin > 12 g/dL	0.12	[0.02; 0.48]	0.002 *

^a^ OR associated with a 10-unit increase. ^b^ OR associated with a 200-unit increase. * *p* < 0.05. Abbreviations: OR: odds ratio, CI95%: 95% confidence interval, LVEF: left ventricle ejection fraction, BNP: B-type natriuretic peptide, CRP: C-reactive protein, GFR: glomerular filtration rate.

## Data Availability

All data generated or analyzed during the study are included in this published article.

## Supplementary Materials

The following supporting information can be downloaded at: https://www.mdpi.com/article/10.3390/jcm12103456/s1, Supplementary Material S1: Supplementary Material to Table 3 in the “Results” section—Specific characteristics related to the occurrence of serious heart rhythm disorder (SHRD).

## Abbreviations

ACS: acute coronary syndrome, STEMI: ST elevation myocardial infarction, NSTE-ACS: non-ST elevation acute coronary syndrome, NSTEMI: non-ST elevation myocardial infarction, SHRD(s): serious heart rhythm disorder(s), ED: emergency department, WHO: World Health Organization, NHC: Nouvel Hôpital Civil, AVB: atrioventricular block, EMD: electromechanical dissociation, VT: ventricular tachycardia, VF: ventricular fibrillation, EMR: electronic medical record, CVRFs: cardiovascular risk factors, ESC: European Society of Cardiology, SXS: SYNTAX score, CSS: clinical SYNTAX score, GFR: glomerular filtration rate, LVEF: left ventricle ejection fraction, AIA: anterior interventricular artery.
