# Supplementary material for: Occurrence of Severe Arrhythmias in Patients with Non-ST Elevation Acute Coronary Syndrome (NSTE-ACS): A Retrospective Study
Source: J Clin Med. 2023 May 14;12(10):3456. doi: 10.3390/jcm12103456 (PMC10219218; doi:10.3390/jcm12103456)
Supplement: Supplementary file 1 [file jcm-12-03456-s001.zip › jcm-2279318-supplementary.pdf]

Supplementary material

Occurrence of Severe Arrhythmias in Patients with Non-ST Elevation Acute Coronary Syndrome (NSTE-ACS): A Retrospective Study

Valérie Wilmé[1*], Sébastien Harscoat[1], François Séverac[2], Adrien Carmona[3], Pierrick Le Borgne[1,4], Pascal Bilbault[1,4], Olivier Morel[3,4] and Sabrina Kepka [1,2,5]

[1] Emergency Department, Hôpitaux Universitaires de Strasbourg, 67091 Strasbourg, France
[2] Public Health Department, Hôpitaux Universitaires de Strasbourg, 67091 Strasbourg, France
[3] Interventional Cardiology Department, Hôpitaux Universitaires de Strasbourg, 67091 Strasbourg, France
[4] French National Institute of Health and Medical Research (INSERM), UMR 1260, Regenerative NanoMedicine (RNM), Fédération de Médecine Translationnelle (FMTS), University of Strasbourg, 67000 Strasbourg, France
[5] ICube, UMR 7357 CNRS, 67400 Illkirch-Graffenstaden, France

S1: Supplementary Material to Table 3 in the "Results" section — Specific characteristics related to the occurrence of serious heart rhythm disorder (SHRD)

Patient N°6 had a history of tritruncal ischemic heart disease, and had acute intra-stent occlusion of the proximal circumflex artery and significant anterior interventricular artery (AIA) stenosis. There was no technical difficulty during angioplasty.

Patient N°8 also had a history of tritruncal ischemic heart disease. Coronary angiography demonstrated an intra-stent subocclusive restenosis of the proximal circumflex artery. Pre- and post-stenting dilatation was difficult, requiring the use of a super high pressure percutaneous transluminal coronary angioplasty balloon.

Patient N°9 had no known history of coronary artery disease. He had a massive calcified tight stenosis of the middle AIA and significant stenosis of the first marginal. There was no difficulty during angioplasty.

Regarding the five events that occurred before coronary angiography (1.0% of the population, CI95% [0.4%; 2.5%]), patient N°1 had a significant hypokalemia which may explain the SHRD and patients N°2 and 5 presented a transient and asymptomatic SHRD that did not require therapeutic measure.

Only two patients who presented an SHRD before coronary angiography required specific management: patient N°3 received a non-invasive temporary cardiac pacing for a poorly tolerated third-degree atrioventricular block and patient N°4 received an external electric shock to reduce ventricular fibrillation. For this patient, there is no mention in the medical record whether or not an external cardiac massage was performed. These two patients represent 0.4% of the study population (IC95% [0.01%; 1.6%]).

For patient N°10, asystole was preceded by episodes of sinus dysfunction with extreme bradycardia (20 to 30 beats/min).

For patient N°8, the ECG before the coronary angiography shows a ST segment depression in lateral leads which was not present at admission.
